# Trifluridine/tipiracil in patients with metastatic gastroesophageal junction cancer: a subgroup analysis from the phase 3 TAGS study

**DOI:** 10.1007/s10120-021-01156-x

**Published:** 2021-03-13

**Authors:** Wasat Mansoor, Hendrik-Tobias Arkenau, Maria Alsina, Kohei Shitara, Peter Thuss-Patience, Sinead Cuffe, Mikhail Dvorkin, David Park, Takayuki Ando, Marc Van Den Eynde, Giordano D. Beretta, Alberto Zaniboni, Toshihiko Doi, Josep Tabernero, David H. Ilson, Lukas Makris, Karim A. Benhadji, Eric Van Cutsem

**Affiliations:** 1 The Christie NHS Foundation Trust, Manchester, UK; 2 Sarah Cannon Research Institute, Cancer Institute, University College London, London, UK; 3 Vall D, Institute of Oncology (VHIO), Hebron University Hospital, Universitat Autònoma de Barcelona, Barcelona, Spain; 4 National Cancer Center Hospital East, Chiba, Japan; 5 Charité-Universitätsmedizin Berlin, Medizinische Klinik M.S. Hämatologie, Onkologie Und Tumorimmunologie, Berlin, Germany; 6 St. James’s Hospital, Dublin, Republic of Ireland; 7 Omsk Regional Clinical Centre of Oncology, Omsk, Russian Federation; 8 St. Jude Crosson Cancer Institute/St, Joseph Heritage Healthcare, Fullerton, CA USA; 9 University of Toyama, Toyama, Japan; 10 UCL Cliniques Universitaires Saint-Luc, Brussels, Belgium; 11 Humanitas Gavazzeni, Bergamo, Italy; 12 Fondazione Poliambulanza–Istituto Ospedaliero, Brescia, Italy; 13 Institute of Oncology (VHIO), Vall D’Hebron University Hospital, UVic-UCC, IOB-Quiron, Barcelona, Spain; 14 Memorial Sloan Kettering Cancer Center, New York, NY USA; 15 Stathmi, Inc, New Hope, PA USA; 16 Taiho Oncology, Inc, Princeton, NJ USA; 17 University Hospitals Gasthuisberg Leuven and KU Leuven, Leuven, Belgium

**Keywords:** Trifluridine/tipiracil, Gastroesophageal junction cancer, TAGS, Phase 3, Subgroup analysis

## Abstract

**Background:**

Patients with advanced gastroesophageal junction cancer (GEJC) have poor survival outcomes, and GEJC-specific data from trials evaluating agents in gastric cancers (GCs) as a whole are lacking. Trifluridine/tipiracil (FTD/TPI) was approved for previously treated metastatic GC or GEJC (mGC/mGEJC) based on results of the phase 3 TAGS trial. Subgroup analyses by primary tumor type (GC or GEJC) in TAGS are reported here.

**Methods:**

Pa tients with mGC/mGEJC treated with  ≥ 2 prior chemotherapy regimens were randomized (2:1) to receive FTD/TPI or placebo, plus best supportive care. A pre-planned sub-analysis was performed to evaluate efficacy and safety outcomes by primary tumor type (GEJC or GC).

**Results:**

Of 507 randomized patients, 145 (29%) had GEJC and 360 (71%) had GC as the primary disease site. Baseline characteristics were generally similar between the GEJC and GC subgroups, except that more patients in the GEJC subgroup had received  ≥ 3 prior regimens (72 vs. 59% in the GC subgroup). Survival benefit with FTD/TPI was observed in both subgroups. The overall survival hazard ratio for FTD/TPI vs placebo was 0.75 (95% CI 0.50–1.11) and 0.67 (95% CI 0.52–0.87) in the GEJC and GC subgroups, respectively. Grade ≥ 3 adverse events of any cause were reported in 75 (77%) and 192 (81%) FTD/TPI-treated patients in the GEJC and GC subgroups, respectively. No new safety concerns were noted with FTD/TPI.

**Conclusion:**

As in patients with GC, FTD/TPI showed an efficacy benefit in patients with GEJC in the TAGS trial, along with demonstrating a manageable safety profile.

**Supplementary Information:**

The online version contains supplementary material available at 10.1007/s10120-021-01156-x.

## Introduction

Gastroesophageal junction cancer (GEJC), though often grouped under gastric cancer (GC) in clinical trial and registries, has distinct clinical features, risk factors, and diagnosis and treatment challenges [1]. The incidence of GEJC has been increasing over several decades, doubling in the United States from 16% in 1973 to 32% in 2013 [2, 3]. GEJC is often diagnosed at a relatively late stage when the disease has become unresectable, and patients with advanced/metastatic GEJC generally require multiple lines of therapy, as recurrence is common [4].

In a real-world analysis of over 3000 patients with advanced GC/GEJC (43% with GEJC); median OS with first-line therapy, composed primarily of chemotherapy combinations, was 10.7 months and declined with each subsequent line of therapy (7.6–2.8 months) [5]. Additional real-world data suggest that patients with GEJC may have reduced landmark survival rates compared with GC at 6 (20 vs. 30%) and 12 months (11 vs. 16%) [6].

Trifluridine/tipiracil (FTD/TPI) is an oral therapy comprising the thymidine analog trifluridine and tipiracil, which prevents trifluridine degradation [7]. FTD/TPI received approval in the United States, Europe, and Japan for previously treated metastatic GC/GEJC based on OS benefit observed in the phase 3 TAGS (TAS-102 Gastric Study; NCT02500043) [8, 9]. Here, we present data from a pre-planned subgroup analysis that was conducted to evaluate the efficacy and safety of FTD/TPI in patients with GEJC.

## Materials and methods

TAGS, a global phase 3 randomized placebo-controlled clinical trial, enrolled patients with non-resectable metastatic GC/GEJC who had received at least two previous chemotherapy regimens and had an Eastern Cooperative Oncology Group performance status (ECOG PS) of 0 or 1. GEJ involvement was documented by endoscopic, radiologic, surgical, or pathology report.

Patients were randomized (2:1) to receive FTD/TPI 35 mg/m^2^ or placebo, both twice daily with best supportive care, on days 1–5 and 8–12 of each 28-day treatment cycle. The primary endpoint was OS; secondary endpoints included progression-free survival (PFS), time to deterioration (TTD) of ECOG PS to  ≥ 2, safety, and tolerability. The protocol was approved by the institutional review board/independent ethics committee at each participating site. The study was conducted in accordance with the Declaration of Helsinki and Good Clinical Practice guidelines. All patients provided written informed consent. Additional details about the conduct of this study have been reported previously [6].

Although pre-planned, the subgroup analyses described in this report were not powered for statistical significance and are not intended to be used to compare results between primary tumor locations with GEJC vs GC involvement. Hazard ratios (HRs) and associated 95% confidence intervals (CIs) for time-to-event endpoints were based on a stratified Cox proportional hazards model; median values were Kaplan–Meier estimates.

## Results

Of 507 patients enrolled in the TAGS trial, 145 (29%) and 360 (71%) had a sole primary tumor location of GEJ or GC, respectively (2 patients had both gastric and GEJC tumors and were excluded from this analysis). Although baseline patient characteristics in these two subgroups were generally similar, there were some notable differences (Table 1). A higher proportion of patients in the GEJC than the GC subgroup were male (85 vs. 68%), were White (83 vs. 65%), had an ECOG PS of 1 (70 vs. 59%), or were more heavily pretreated (72 vs. 59% completing ≥ 3 prior regimens). Within both subgroups, baseline characteristics were generally similar between the treatment groups, with some exceptions in the GEJC subgroup. In this subgroup, patients randomized to FTD/TPI versus placebo were more heavily pretreated (74 vs. 66% had received ≥ 3 prior regimens), and a smaller proportion had undergone prior gastrectomy (40 vs. 55%).

**Table 1:** Baseline clinical and disease characteristics

	GEJC^a^		GC^a^	
	FTD/TPI	Placebo	FTD/TPI	Placebo
	(*n* = 98)	(*n* = 47)	(*n* = 239)	(*n* = 121)
Age, years				
Mean	61	62	63.4	61.9
Median (range)	62.0 (24–89)	62.0 (42–80)	64 (27–86)	63 (32–82)
Sex, *n* (%)				
Male	83 (85)	40 (85)	169 (71)	76 (63)
Race, *n* (%)				
White	83 (85)	37 (79)	161 (67)	74 (61)
Asian	6 (6)	4 (9)	45 (19)	25 (21)
Black	0	0	1 (< 1)	2 (2)
Not collected	8 (8)	4 (9)	30 (13)	20 (17)
Other	1 (1)	2 (4)	2 (1)	0
ECOG PS, *n* (%)				
0	28 (29)	15 (32)	95 (40)	53 (44)
1	70 (71)	32 (68)	144 (60)	68 (56)
Geographic region, *n* (%)				
Japan	6 (6)	4 (9)	40 (17)	23 (19)
USA	13 (13)	3 (6)	8 (3)	2 (2)
EU	79 (81)	40 (85)	191 (80)	96 (79)
Previous gastrectomy, *n* (%)				
Yes	39 (40)	26 (55)	108 (45)	156 (40)
No	59 (60)	21 (45)	131 (55	73 (60)
Prior radiotherapy, *n* (%)				
Yes	36 (37)	17 (36)	35 (15)	9 (7)
No	62 (63)	30 (64)	204 (85)	112 (93)
Number of metastatic sites, *n* (%)				
1–2	50 (52)	25 (53)	105 (44)	47 (39)
≥ 3	48 (49)	22 (47)	134 (56)	74 (61)
Number of prior regimens, *n* (%)				
2	25 (26)	16 (34)	101 (42)	47 (39)
3	41 (42)	15 (32)	93 (39)	45 (37)
≥ 4	32 (33)	16 (34)	45 (19)	29 (24)

*EU* Europe, *FTD/TPI* trifluridine/tipiracil, *GEJC* gastroesophageal junction cancer, *GC* gastric cancer, *USA* United States of America

^a^Two patients had both gastric and GEJC tumors and were excluded from this analysis

At data cutoff (31 March 2018),  ≥ 94% of patients in both treatment arms in each tumor-type subgroup had discontinued treatment (Supplementary Table). The most common reason for discontinuation in both the GEJC and GC subgroups was disease progression (78% of GEJC and 72% of GC in FTD/TPI-treated arm; GEJC of 87% and 86% of GC in placebo-treated arm).

**Fig. 1:**
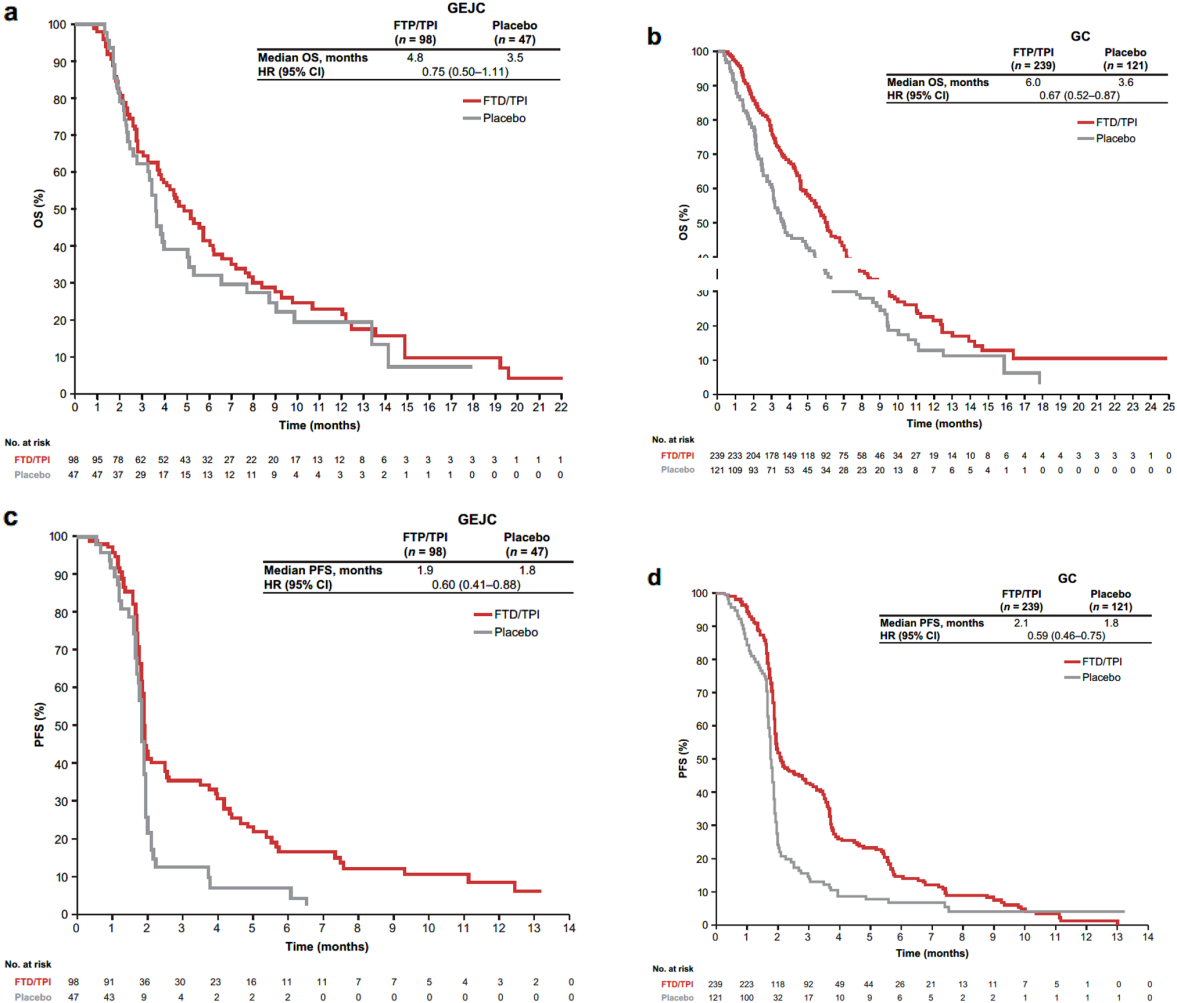
Efficacy outcomes in the GEJC and GC subgroups. **a** OS in the GEJC subgroup. **b** OS in the GC subgroup. **c** PFS in the GEJC subgroup. **d** PFS in the GC subgroup. CI: confidence interval, ECOG PS: Eastern Cooperative Oncology Group performance status, GEJC: gastroesophageal junction cancer, GC: gastric cancer, HR: hazard ratio, PFS: progression-free survival, OS: overall survival

**Fig. 2:**
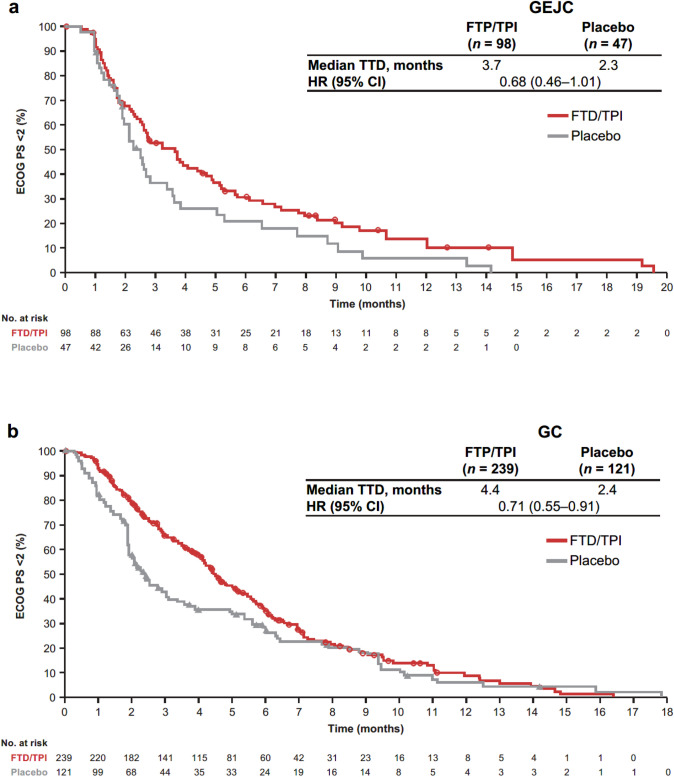
Time to deterioration in the GEJC and GC subgroups. **a** TTD of ECOG PS to ≥ 2 in the GEJC subgroup. **b** TTD of ECOG PS to ≥ 2 in the GC subgroup. CIL: confidence interval, ECOG PS: Eastern Cooperative Oncology Group performance status, GEJC: gastroesophageal junction cancer, GC: gastric cancer, HR: hazard ratio, TTD: time to deterioration

In both the GEJC and GC subgroups, efficacy outcomes were improved with FTD/TPI compared with placebo (Fig. 1). In the GEJC subgroup, OS and PFS HRs were 0.75 (95% CI 0.50–1.11) and 0.60 (95% CI 0.41–0.88), respectively. In the GC subgroup, OS and PFS HRs were 0.67 (95% CI 0.52–0.87) and 0.59 (95% CI 0.46–0.75). Median OS in the FTD/TPI group was numerically lower in the GEJC than the GC subgroup (4.8 vs. 6.0 months). The HR for TTD of ECOG PS to  ≥ 2 for FTD/TPI vs placebo was 0.68 (95% CI 0.46–1.01) in the GEJC subgroup and 0.71 (95% CI 0.55–0.91) in the GC subgroup (Fig. 2).

Grade ≥ 3 adverse events (AEs) of any cause with FTD/TPI were reported in 75 (77%) and 192 (81%) patients in the GEJC and GC subgroups, respectively (Table 2). The most frequently reported grade ≥ 3 AEs with FTD/TPI in the GEJC group were neutropenia (25%) and anemia (13%); incidences of these AEs in the GC subgroup were 38 and 21%, respectively. In the GEJC subgroup, dosing modifications and discontinuations due to AEs of any cause with FTD/TPI were 41 (42%) and 7 (7%), respectively, and in the GC subgroup, were 107 (45%) and 29 (12%). Treatment-related deaths were reported in 1 (< 1%) FTD/TPI-treated patient (attributed to cardiopulmonary arrest) and 1 (1%) placebo-treated patient (attributed to toxic hepatitis), both in the GC subgroup.

**Table 2:** Adverse events

	GEJC				GC			
	FTD/TPI (*n* = 97)^a^		Placebo (*n* = 46)^a^		FTD/TPI (*n* = 238)^a^		Placebo (*n* = 120)^a^	
	Any grade	Grade ≥ 3	Any grade	Grade ≥ 3	Any grade	Grade ≥ 3	Any grade	Grade ≥ 3
	*n* (%)	*n* (%)	*n* (%)	*n* (%)	*n* (%)	*n* (%)	*n* (%)	*n* (%)
Any AE of any cause	96 (99)	75 (77)	44 (96)	27 (59)	230 (97)	192 (81)	111 (93)	69 (58)
Any treatment-related AE	73 (75)	40 (41)	23 (50)	2 (4)	198 (83)	136 (57)	71 (59)	19 (16)
Action taken due to AEs of any cause								
Dosing modification (dosing delay or dose reduction)	52 (54)	41 (42)	11 (24)	9 (20)	143 (60)	107 (45)	25 (21)	19 (16)
Treatment discontinuation	9 (9)	7 (7)	5 (11)	3 (7)	34 (14)	29 (12)	23 (20)	18 (15)
AEs of any cause in ≥ 10% of patients								
Hematologic AEs								
Neutropenia^b^	41 (42)	24 (25)	0	0	135 (57)	90 (38)	7 (6)	0
Anemia^c^	36 (37)	13 (13)	6 (13)	2 (4)	114 (48)	51 (21)	25 (21)	10 (8)
Leukopenia^d^	16 (17)	1 (1)	0	0	62 (26)	30 (13)	3 (3)	0
Thrombocytopenia^e^	12 (12)	1 (1)	0	0	48 (20)	10 (4)	8 (7)	0
Gastrointestinal AEs								
Nausea	43 (44)	5 (5)	13 (28)	1 (2)	81 (34)	5 (2)	40 (33)	4 (3)
Vomiting	26 (27)	4 (4)	11 (24)	0	57 (24)	8 (3)	22 (18)	3 (3)
Diarrhea	22 (23)	2 (2)	6 (13)	1 (2)	54 (23)	7 (3)	17 (14)	2 (2)
Abdominal pain	19 (20)	4 (4)	10 (22)	7 (15)	36 (15)	10 (4)	21 (18)	8 (7)
Ascites	4 (4)	1 (1)	0	0	15 (6)	11 (5)	16 (13)	11 (9)
Constipation	27 (28)	3 (3)	12 (26)	2 (4)	18 (8)	1 (< 1)	13 (11)	2 (2)
Dysphagia	15 (15)	5 (5)	4 (9)	3 (7)	5 (2)	2 (1)	3 (3)	1 (1)
Other AEs								
Decreased appetite	28 (29)	5 (5)	15 (33)	2 (4)	87 (37)	24 (10)	36 (30)	9 (8)
Fatigue	35 (36)	10 (10)	11 (24)	0	54 (23)	13 (6)	23 (19)	10 (8)
Asthenia	18 (19)	4 (4)	8 (17)	1 (2)	47 (20)	12 (5)	32 (27)	10 (8)
Back pain	8 (8)	1 (1)	5 (11)	3 (7)	17 (7)	1 (< 1)	6 (5)	1 (1)
Dyspnea	12 (12)	5 (5)	5 (11)	1 (2)	12 (5)	1 (< 1)	12 (10)	5 (4)
General physical health deterioration	9 (9)	8 (8)	4 (9)	4 (9)	14 (6)	14 (6)	12 (10)	10 (8)

*AE* adverse event, *FTD/TPI* trifluridine/tipiracil, *GEJC* gastroesophageal junction cancer, *GC* gastric cancer

^a^As treated population

^b^Neutropenia and/or decreased neutrophil count

^c^Anemia and/or decreased hemoglobin level

^d^Leukopenia and/or decreased white blood cell count

^e^Thrombocytopenia and/or decreased platelet count

## Discussion

This subgroup analysis of the TAGS trial provides detailed efficacy and safety data in patients with metastatic GEJC treated with FTD/TPI. The analysis demonstrated efficacy benefits with FTD/TPI in both the GEJC and GC subgroups.

In multivariate Cox regression analyses of OS in TAGS, which included stratification factors and primary tumor site, primary tumor site (gastric or GEJ) was not identified as being prognostic or predictive of OS with FTD/TPI treatment (*P*_interaction_ = 0.29). In the current analysis, median OS with FTD/TPI was marginally lower in the GEJC (4.8 months) than the GC subgroup (6.0 months), although OS was similar with placebo in both subgroups (3.5 and 3.6 months). This could be attributed to patients in the GEJC subgroup overall being more heavily pretreated overall (72 vs. 58% of patients in the GC subgroup having received ≥ 3 previous lines of therapy), as well as differences in the proportion of patients receiving ≥ 3 prior lines treatment between FTD/TPI-treated (74%) and placebo-treated patients (66%) within the GEJC subgroup.

To date, data in GEJC subgroups in trials of other anticancer agents have been limited to mostly HRs of survival, with few studies reporting survival data. The KEYNOTE-059 study, one of the few with survival data, reported similar median OS in the GEJC and GC subgroups (5.7 months [95% CI 4.2–8.4) and 5.6 months [3.8–7.2]) in the GC subgroups with pembrolizumab [10]. OS HRs for the GEJC and GC subgroups reported in other phase 3 studies, such as KEYNOTE-061 (pembrolizumab vs paclitaxel; 0.61 [0.41–0.90] and 0.94 [0.71–1.23] for GEJC and GC), ATTRACTION-2 (nivolumab vs placebo; 0.44 [0.20–0.97] and 0.69 [0.55–0.87], respectively) and RAINBOW (ramucirumab plus paclitaxel vs placebo plus paclitaxel; 0.52 [0.35–0.78] and 0.90 [0.70–1.10], respectively) each indicated a marginally greater death risk reduction with the investigational regimen in the GEJC subgroup than in the GC subgroup [11–13]. In contrast, earlier trials testing non-immune-related agents showed trends towards better survival outcomes in the GC subgroup. For example, the ToGA trial in which the location of the primary cancer was stratified for reported OS HRs for GEJC and GC for chemotherapy/ trastuzumab versus chemotherapy as 0.67 (95% CI 0.42–1.08) vs. 0.76 (95% CI 0.60–0.96), respectively [14]. Possible mechanism for why GEJC does better or worse than GC is difficult based on the current evidence base. Many earlier trials were not stratified for the two anatomical sites, thus, making safe comparative conclusions difficult. There are differences in molecular characteristics between GEJC and GC as identified in the Cancer Genome Atlas (TGCA) which may explain differences in responsiveness to cancer [14]. As discussed, studies testing the emerging immune checkpoint inhibitors may demonstrate a clearer difference in survival outcomes predicated on the molecular differences of the two anatomical sites [15].

In the current sub-analysis, no new safety concerns were noted with FTD/TPI in the GEJC subgroup, and the incidence of grade ≥ 3 hematologic AEs appeared to be lower than in the FTD/TPI-treated GC subgroup. Comparable safety data have not been reported by these subgroups in trials of other agents, including those mentioned above.

The main limitation of the current analyses was that although they were pre-planned, they were not powered for statistical significance. This precluded a robust evaluation of the efficacy and safety of FTD/TPI in the GEJC or GC subgroups.

## Conclusion

In summary, the results of this analysis indicate that FTD/TPI is an effective treatment option with a manageable safety profile in patients with metastatic GEJC, similar to what was observed in GC. FTD/TPI resulted in an efficacy benefit in the GEJC subgroup despite patients in the FTD/TPI group being more heavily pretreated than in the placebo group.

## Supplementary Information

Below is the link to the electronic supplementary material.

Supplementary file1 (DOCX 15 KB)
