# Supplementary material for: Trifluridine/tipiracil in patients with metastatic gastroesophageal junction cancer: a subgroup analysis from the phase 3 TAGS study
Source: Gastric Cancer. 2021 Mar 13;24(4):970–7. doi: 10.1007/s10120-021-01156-x (PMC8205879; doi:10.1007/s10120-021-01156-x)
Supplement: Supplementary file 1 — Supplementary file1 (DOCX 15 KB) [file 10120_2021_1156_MOESM1_ESM.docx]

**Online Supplementary Appendix**

**Supplementary Table** Patient disposition

|  | **GEJC** | | **GC** | |
| --- | --- | --- | --- | --- |
|  | **FTD/TPI**  **(*n* = 97)^a^**  ***n* (%)** | **Placebo**  **(*n* = 46)^a^**  ***n* (%)** | **FTD/TPI**  **(*n* = 238)^a^**  ***n* (%)** | **Placebo**  **(*n* = 120)^a^**  ***n* (%)** |
| Ongoing | 5 (5) | 0 | 14 (6) | 3 (3) |
| Discontinued study treatment  Radiologic/clinical progression  Adverse event  Death^b^  Other | 92 (95)  75 (78)  10 (10)  3 (3)  4 (4) | 46 (100)  40 (87)  2 (4)  1 (2)  3 (7) | 224 (94)  171 (72)  23 (10)  8 (3)  23 (10) | 117 (98)  103 (86)  9 (8)  1 (1)  4 (3) |

*FTD/TPI* trifluridine/tipiracil, *GEJC* gastroesophageal junction cancer, *GC*, gastric cancer

^a^As-treated population

^b^There were 2 treatment-related deaths: 1 due to cardiorespiratory arrest in the FTD/TPI group, and 1 due to toxic hepatitis in the placebo group; both patients had GC
